# Laparoscopic resection of two inflammatory fibroid polyps: An unusual cause of jejunojejunal intussusception

**DOI:** 10.1016/j.ijscr.2020.03.029

**Published:** 2020-03-28

**Authors:** Ki Bum Park, Ye Seob Jee, Dong-Wook Kim

**Affiliations:** a Department of Surgery, School of Medicine, Kyungpook National University, Daegu, Republic of Korea; b Department of Surgery, Dankook University Hospital, Chungnam, Republic of Korea

**Keywords:** Inflammatory fibroid polyp, Intussusception, Laparoscopic surgery

## Abstract

**Introduction:**

Inflammatory fibroid polyps (IFPs) are rare, clinically benign tumors that can present as an intussusception causing intestinal obstruction.

**Presentation of case:**

We present a rare case of a 23-year-old man who suffered from intussusception caused by two IFPs. The patient complained of aggravated squeezing epigastric pain that prompted him to visit the emergency department. Moderate abdominal distention with mild tenderness was noted without signs of peritonitis. Abdominal computed tomography confirmed the presence of intussusception in the left lower quadrant. During laparoscopic surgery, the intussuscepted segment of the jejunum was identified, after which laparoscopic small bowel resection and anastomosis were performed. The patient was subsequently discharged without postoperative complications.

**Discussion:**

IFPs are rare, clinically benign tumors of the gastrointestinal tract originating from the submucosa. IFPs can present as an intussusception causing intestinal obstruction.

**Conclusion:**

Surgical resection is the optimal treatment method for IFPs accompanied by intussusception.

## Highlights

• IFPs are rare, clinically benign tumors that can present as an intussusception.
• 23-year-old man suffered from intussusception caused by two IFPs.
• Surgical resection may be the optimal treatment method for IFPs accompanied by intussusception.

## 1 Introduction

Intussusception is defined as the invagination of a proximal portion of the gut into a distal portion [1]. Intussusception is relatively rare among adults (approximately 5% of total intussusception), with a leading point in the form of a benign mass usually being identified. Inflammatory fibroid polyps (IFPs), also known as Vanek’s tumors, are benign submucosal lesions of the gastrointestinal tract [2,3]. Although IFPs in the small intestine are commonly asymptomatic, symptoms can develop depending on the size and location of the tumor. Here, we present a rare case involving a 23-year-old man who suffered from intussusception caused by IFPs. We subsequently review the relevant literature and highlight the efficiency of laparoscopic surgery for treatment of this disease. This work has been reported in line with the SCARE criteria [4].

## 2 Case report

A 23-year-old man visited the emergency department with a 3-day history of diffuse abdominal pain. The patient complained of aggravated squeezing epigastric pain every 3 min. Furthermore, he suffered from constipation, nausea, and vomiting for the past 2 days. The patient had no history of fever, hematochezia, and weight loss and had an unremarkable drug, family, or surgical history.

On physical examination, vital signs, including blood pressure, heart rate, body temperature, respiratory rate, and oxygen saturation at room air, were normal. Moderate abdominal distention with mild tenderness was noted without signs of peritonitis. The patient did not exhibit guarding or rebound tenderness. An erect abdominal X-ray showed a stepladder sign, suggesting mechanical obstruction. Laboratory examination results, including blood chemistry, routine blood tests, and tumor markers, were normal.

**Fig. 1:**
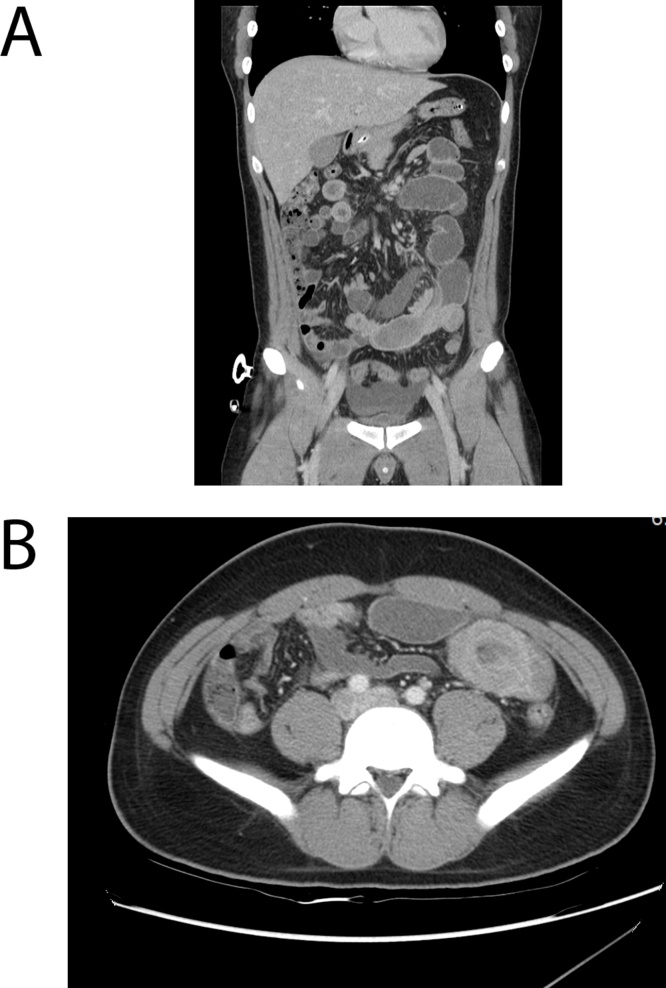
Computed tomography revealing intussusception. (A) coronal view; (B) axial view.

**Fig. 2:**
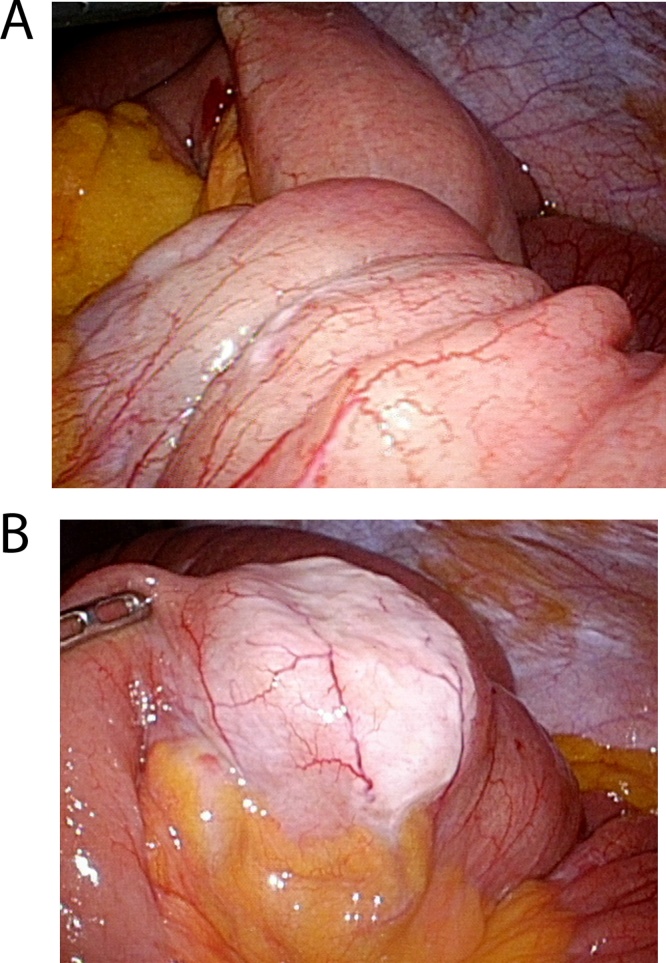
Laparoscopic intraoperative view. (A) Intussuscepted portion of the jejunum; (B) lead point presumably indicating a mass-like lesion.

The patient was first admitted to the internal medicine division of gastroenterology. A nasogastric tube was then immediately inserted for gastrointestinal decompression, through which more than 500 mL of a greenish, foul-smelling fluid was drained per day. Two days later, abdominal computed tomography (CT) confirmed the presence of intussusception in the left lower quadrant (Fig. 1). No mass-like lesion or lead point could be clearly detected on the CT scan. He was transferred to the general surgery department and offered laparoscopic surgery for the purpose of small bowel reduction and segmental resection. A 12-mm port for the 3-dimensional scope was inserted into the umbilical site, while two 5-mm ports were inserted into the right upper abdomen. Precise laparoscopic examination was performed, through which an intussuscepted segment of the jejunum 120 cm from the ligament of Treitz was detected (Fig. 2A). The rest of the abdominal viscera were normal. After performing laparoscopic bowel reduction, a mass-like lesion was identified (Fig. 2B). Wide excision of the jejunum and side-to-side anastomosis were performed through laparoscopic technique using a linear stapler. The patient was then transferred to the general ward after surgery and tolerated the procedure well. He was discharged on the fifth postoperative day without immediate complications. A follow-up appointment after discharge revealed that the patient was in good condition without any symptoms.

**Fig. 3:**
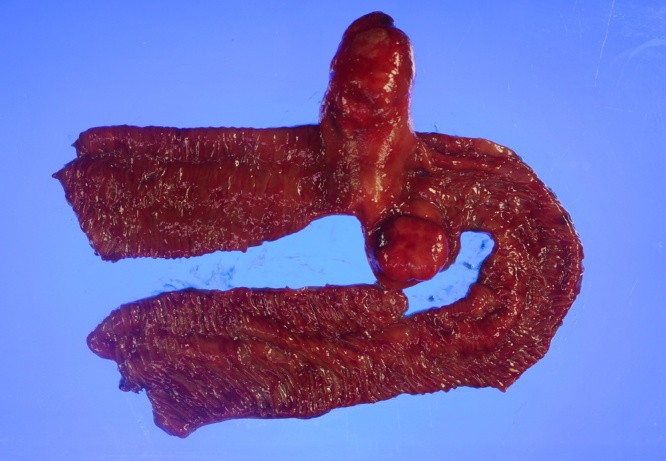
Two polypoid lesions along with the resected jejunal segment.

**Fig. 4:**
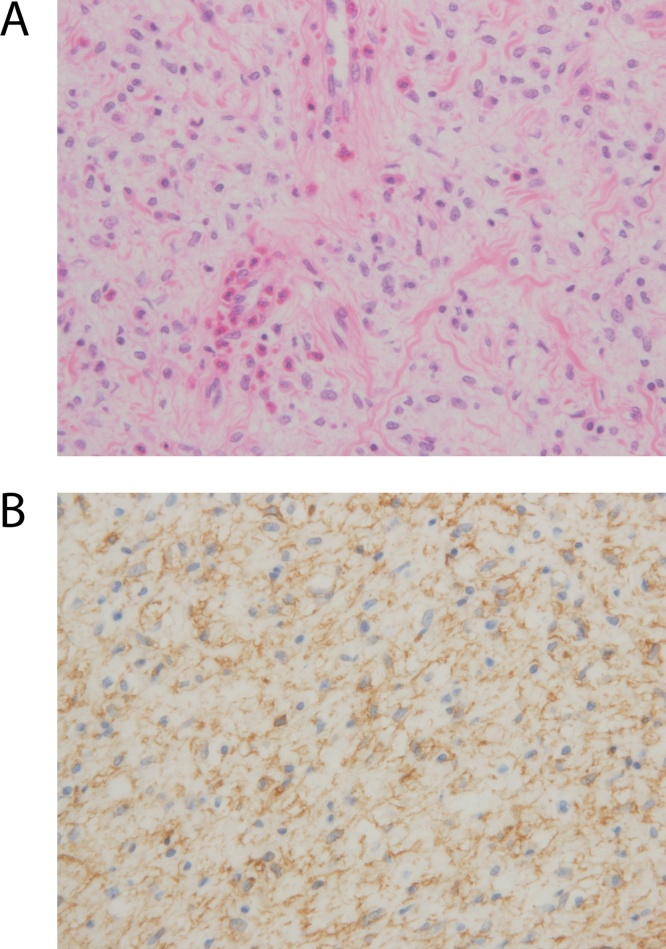
Photomicrograph of the jejunal polyp. (A) H&E staining; (B) positive immunostaining for CD34.

Macroscopic findings of the resected specimen revealed two intraluminal polyps measuring 5 × 3 × 3 cm and 2.5 × 2.3 × 1.2 cm that originated from the same location. The larger one had a pedunculated stalk, whereas the small one had a sessile stalk (Fig. 3). Cross section of the two polyps revealed a grayish white, solid, homogeneous, and slightly glistening cut surface. The remaining intestinal mucosa was grossly unremarkable albeit edematous. Microscopic examination of the mass revealed a CD34-positive (Fig. 4A) submucosal lesion showing a mixture of spindle cells, inflammatory cells, and prominent capillaries, which was subsequently determined to be IFP (Fig. 4B). Immunohistochemical results revealed CD117 (−), bcl-2 (−), desmin (−), SMA (−), S-100 (−), and a Ki-67 positivity rate of 1%–5%.

## 3 Discussion

Intussusception was first reported by Barbette in 1674, while Jonathan Hutchinson performed the first surgical intervention in 1871 [5]. This condition occurs as a result of motility disorder between two segments of the intestine. While primary intussusception is common among children, approximately 80%–90% of adult cases are secondary to a lead point such as benign or malignant tumors. Small bowel intussusception is usually caused by benign lesions, including lipomas, adhesion, hamartomas, Meckel’s diverticulum, and several types of polyps [6]. The ileal segment of the small bowel is the most common location for intussusception, though some rare cases may develop in the jejunum. Intussusception causes bowel obstruction symptoms, including nausea, vomiting, and episodic abdominal pain, with abdominal CT being the preferred diagnostic tool for adults. The typical CT findings in intussusception are an unhomogeneous “target” shaped soft tissue mass with a layering effect [7]. Once diagnosed, surgical treatment is needed to prevent complications, such as bowel necrosis, perforation, and ischemia.

IFPs are known by various names, including Vanek’s tumor, polypoid myoendothelioma, inflammatory pseudotumor, and eosinophilic granuloma [8]. They are rare, clinically benign tumors of the gastrointestinal tract originating from the submucosa. IFP affects usually in the 5th to 7th decade of life, with a slight female predominance [5]. They have been most commonly found in the gastric antrum (60%–70%), followed by the small bowel (18%–20%), colon (4%–7%), and duodenum, gallbladder, and esophagus (1%) [9]. Though the etiology of IFPs has remained unknown, studies have suggested trauma, allergic reaction, and bacterial and chemical irritation as possible factors. Polyps typically present as solitary masses measuring 0.2–20 cm in diameter that project into the bowel lumen. They are typically characterized by fibroblastic and vascular proliferation with extensive eosinophilic infiltration on microscopy. On immunohistochemistry, IFPs show positive staining for CD34 and negative staining for S100 and CD117, allowing for differentiation from gastrointestinal stromal tumors [10].

IFPs are usually found incidentally during endoscopy, imaging investigations, and surgery. Clinical aspects depend largely on the tumor size and location. Even if there is no obstruction or intussusception symptoms, IFPs become apparent with gastrointestinal bleeding caused by superficial mucosal erosion or ulceration [11]. Though often asymptomatic, colicky abdominal pain, nausea, vomiting, and abdominal distension can occur when this tumor cause bowel obstruction and intussusception. In our case, the patient decided not to immediately visit the hospital despite having a history of diffuse abdominal pain for 3 days.

Radiological findings can be helpful in diagnosing IFP-related intussusception. Generally, abdominal X-ray is the first diagnostic tool used to detect bowel obstruction. Ultrasonography has also been considered an effective tool in the diagnosis of intussusception, with imaging features characterized by a target or ‘donut sign’. Ultrasonography has a sensitivity of 98%–100% and a specificity of 88%–89% [2]. Abdominal CT has been the most accurate diagnostic method for confirming intussusception. However, visualization of a mass within the intussusception is rare, as shown in the present case. Differential diagnoses for IFPs include malignancy, lipomas, neurofibromas, foreign bodies, leiomyomas, gastrointestinal stromal tumors, inflammatory bowel disease, and several other tumors that can cause intussusception [2].

Although surgical resection has been the primary treatment method for IFPs, those that occur in stomach and duodenum can be removed through endoscopy. IFPs are usually benign and neither recur nor metastasize. However, given that establishing a diagnosis of IFP through preoperative imaging alone is difficult, the possibility of malignancy should be considered. In our case, we performed wide resection of the intussuscepted segment including the two IFPs. Given the possibility for malignancy in the gross appearance, extensive small bowel and mesentery resection with margins of at least 50 mm from the adjacent tissue should be performed. Regarding the technical aspect, this operation was performed using the laparoscopic technique without a first assistant. Currently, minimally invasive surgical approaches to abdominal surgery have been gradually replacing conventional open surgery considering the several advantages they offer, including reduced postoperative pain and better cosmetic outcomes [12]. Given that the patient’s physical and radiologic findings showed no signs of ischemia or necrosis of the intestine, we were able to perform laparoscopic surgery more easily.

To the best of our knowledge, this case, in which two IFPs originated from the same location in the jejunum, is very rare. In summary, IFPs are rare, clinically benign tumors that can present as an intussusception causing intestinal obstruction. Surgical resection is the optimal treatment method for IFPs accompanied by intussusception since these lesions do not metastasize and have no recurrence rate.

## Declaration of Competing Interest

There are no potential conflicts of interest relevant to this article.

## Funding

There are no sources of funding relevant to this article.

## Ethical approval

The institutional review board of Dankook University Hospital approved the protocol (IRB number; 202001013).

## Consent

Written informed consent was obtained from the patient.

## Author contribution

Concept: K.D.W.

Data collection: P.K.B.

Data analysis: J.Y.S.

Writing papers: P.K.B, K.D.W.

Supervision: K.D.W.

## Registration of research studies

None.

## Guarantor

Dong-Wook Kim is the guarantor.

## Provenance and peer review

Not commissioned, externally peer-reviewed.
